# Unraveling the karyotype structure of the spurges *Euphorbia hirta* Linnaeus, 1753 and *E. hyssopifolia* Linnaeus, 1753 (Euphorbiaceae) using genome size estimation and heterochromatin differentiation

**DOI:** 10.3897/CompCytogen.v10i4.8193

**Published:** 2016-12-01

**Authors:** Karla C. B. Santana, Diego S. B. Pinangé, Santelmo Vasconcelos, Ana R. Oliveira, Ana C. Brasileiro-Vidal, Marccus V. Alves, Ana M. Benko-Iseppon

**Affiliations:** 1 Departamento de Genética, Centro de Ciências Biológicas, Universidade Federal de Pernambuco, Av. da Engenharia s/n, CEP 50740-600 Recife, PE, Brazil; 2 Departamento de Botânica, Centro de Ciências Biológicas, Universidade Federal de Pernambuco, Rua Prof. Nelson Chaves s/n, CEP 50670-901 Recife, PE, Brazil; 3 Department of Sustainable Development, Vale Institute of Technology, Rua Boaventura da Silva, 955, Umarizal, CEP 66055-090, Belém, PA, Brazil

**Keywords:** Cytotaxonomy, CMA/DAPI, FISH, genome size, rDNA, RONs

## Abstract

*Euphorbia* Linnaeus, 1753 (Euphorbiaceae) is one of the most diverse and complex genera among the angiosperms, showing a huge diversity in morphologic traits and ecologic patterns. In order to improve the knowledge of the karyotype organization of *Euphorbia hirta* (2n = 18) and *Euphorbia hyssopifolia* (2n = 12), cytogenetic studies were performed by means of conventional staining with Giemsa, genome size estimations with flow cytometry, heterochromatin differentiation with chromomycin A_3_ (CMA) and 4’,6-diamidino-2-phenylindole (DAPI) and Giemsa C-banding, fluorescent *in situ* hybridization (FISH) with 45S and 5S rDNA probes, and impregnation with silver nitrate (AgNO_3_). Our results revealed small metacentric chromosomes, CMA^+^/DAPI^0^ heterochromatin in the pericentromeric regions of all chromosomes and CMA^+^/DAPI^−^ in the distal part of chromosome arms carriers of nucleolar organizing regions (NORs). The DNA content measurements revealed small genomes for both species: *Euphorbia hirta* with 2C = 0.77 pg and *Euphorbia hyssopifolia* with 2C = 1.41 pg. After FISH procedures, *Euphorbia hirta*, and *Euphorbia hyssopifolia* presented three and four pairs of terminal 45S rDNA sites, respectively, colocalizing with CMA^+^ heterochromatic blocks, besides only one interstitial pair of 5S rDNA signals. Additionally, the maximum number of active NORs agreed with the total number of observed 45S rDNA sites. This work represents the first analysis using FISH in the subfamily Euphorbioideae, revealing a significant number of chromosomal markers, which may be very helpful to understand evolutionary patterns among *Euphorbia* species.

## Introduction

The giant genus *Euphorbia* (spurges), a member of the family Euphorbiaceae, is one of the largest and most diverse groups of the plant kingdom, consisting of more than 2000 species with a very wide geographic distribution (Bruyns et al. 2006, The Plant List 2013, Webster 2014). The species of the family are used mainly for ornamental and/or medicinal purposes (e.g. Shi et al. 2008, Mwine and Van Damme 2011), although some caution must be required due to the toxic potential of these plants (e.g. Araújo et al. 2015). *Euphorbia hirta* and *Euphorbia hyssopifolia* are cosmopolitan spurges with widely known medicinal properties, standing out mainly due to their diuretic and antimicrobial activities (e.g. Ayyannar and Ignacimuthu 2009, Alisi and Abanobi 2012, Huang et al. 2012, Kuta et al. 2014, Santana et al. 2015). Both species are sub-spontaneous and ruderal, native to the New World, tolerant to drought and high temperatures (Steinmann and Porter 2002). They are broadly distributed in subtropical and tropical regions, from the sea level up to 1500 m (Amorozo 2002, Schneider 2007). In Brazil, they are often sympatric (Santana et al. 2015) and occur in all regions and biomes, where they inhabit degraded areas, roadsides, cultivated fields and gardens (Steinmann and Porter 2002).

Similarly to the family as a whole, the genus *Euphorbia* is an extremely diversified group, not only taking into account morphology and habit (Webster 1994) but also regarding karyotypic characters (Hans 1973). Therefore, the vast complexity of the genus may explain the controversies among the few analyses of the phylogenetic relationships within the group (see Bruyns et al. 2006, Horn et al. 2012, Dorsey et al. 2013). According to Bruyns et al. (2006), the development of a natural classification for *Euphorbia* has been hampered by several factors, such as the high number of species, the wide geographic distribution of the genus and a high degree of convergence in various vegetative characters.

In groups with such a complex classification, the knowledge about chromosome features, such as the organization of interphase nuclei, diploid number, nuclear DNA content and physical mapping of repetitive DNA, may be critical to support studies on systematics and understanding evolutionary pathways (Benko-Iseppon and Morawetz 2000, Guerra 2012). However, to date, just a few studies have provided some help towards elucidating the karyotypic patterns for both the genus and the family. As mentioned by D’Emerico et al. (2003), the available karyotype data for *Euphorbia* species are minimal and, in most cases, there are only descriptions of chromosome numbers. Thus, aiming to increase the cytogenetic data and to identify chromosome markers for this important genus, cytogenetic analyses were performed with conventional staining, genome size estimations through flow cytometry, Giemsa C-banding, CMA/DAPI banding, impregnation with silver nitrate, FISH with 45S and 5S rDNA probes in the species *Euphorbia hirta* and *Euphorbia hyssopifolia*.

## Material and methods

Fruits of specimens of *Euphorbia hirta* (vouchers: K.C.B. Santana 04, 05 and 06 – UFP) and *Euphorbia hyssopifolia* (vouchers: K.C.B. Santana 01, 02 and 03 – UFP) were collected in urban fragments of the Atlantic Forest in Recife (Pernambuco, Brazil). Subsequently, they were incubated at 50 °C for 5 h and then transferred to room temperature (ca. 25 °C) for three to four days to release the seeds, which were germinated in Petri dishes under an artificial system of circadian lighting (≥ 1,500 lux) at ~35 °C. Root tips were pre-treated with 2 mM 8-hydroxyquinoline for 90 min at room temperature and 23 h at 8 °C. For the conventional staining, fluorochromes and FISH procedures, the roots were fixated in ethanol:acetic acid (3:1, v:v), for 4–6 h at room temperature and stored at −20 °C.

The preparation of slides followed the methodology used by Benko-Iseppon and Morawetz (2000). Root tips were hydrolyzed in 5N HCl for 20 min at room temperature and squashed in 45% acetic acid. Slides were stained with 2% Giemsa for 10 min, washed with distilled water and mounted with Entellan (Merck).

To estimate the DNA C-values, approximately 20-30 mg of fresh leaves from *Euphorbia hirta* and *Euphorbia hyssopifolia* were chopped on ice with 1 mL of GPB buffer (Loureiro et al. 2007), with the addition of 3% PVP and 4% Triton X-100, to release the nuclei, according to Dolezel et al. (1989), using *Solanum lycopersicum* Linnaeus, 1753 (2C = 2.06 pg) as the internal reference standard. For each species, three different samples were prepared, and at least 5,000 nuclei were analyzed for each species using a BD FACSAria II (BD Biosciences, San Jose, CA, USA) cytometer. Each output flow cytometry histogram from BD FACSDiVa software v. 6.1 was analyzed using Flowing Software v. 2.5 by Perttu Terho (Turku Centre for Biotechnology, University of Turku, Turku, Finland), with all peaks presenting a coefficient of variation smaller than 4%. The DNA 2C-values of each sample were calculated by the relative fluorescence intensity of the sample and the internal reference standard.

The C-banding methodology followed the procedures described by Guerra and Souza (2002), with some modifications. Slides were immersed in preheated 45% acetic acid at 60 °C for 30 min, followed by washing in preheated distilled water (at 60 °C) that was gradually changed by water at room temperature. After drying, the slides were incubated in 5% Ba(OH)_2_ for 30 min at room temperature and then washed with distilled water. Afterward, slides were immersed in a 2×SSC solution (300 mM NaCl and 30 mM Na_3_C_6_H_5_O_7_.2H_2_O) for 2 h at 60 °C, being subsequently washed with distilled water. The fluorochrome staining was performed as described below.

The impregnation with silver nitrate followed the protocol described by Vieira et al. (1990), with modifications implemented by Vasconcelos et al. (2010). After pretreatment procedures, roots were fixated in 50% ethanol, acetic acid and 37% formaldehyde (18:1:1, v/v/v) for 4 h at room temperature. Fixated roots were washed with distilled water and then incubated in an aqueous solution of 20% silver nitrate at 60 °C for at least 12 h. After removal of silver residues, the staining was revealed in a solution of 1% hydroquinone in 10% formaldehyde (1:1, v/v) for 2 min, followed by washing with distilled water. The meristematic tissue was squashed between slide and coverslip in 45% acetic acid with a drop of 1% acetic carmine. Then, the slides were frozen in liquid nitrogen, immersed in absolute ethanol for 4 min, dried and mounted with Entellan.

The CMA/DAPI banding followed Schweizer and Ambros (1994), with some modifications. Root tips were washed three times (5 min each) in distilled water and digested for 2 h at 37 °C in an enzymatic solution of 2% cellulase (Onozuka) and 20% pectinase (Sigma). Meristems were washed, placed on slides and then squashed in 45% acetic acid. Chromosome preparations were aged for three days at room temperature, stained with CMA (0.5 mg/mL) for 1 h and DAPI (2 µg/mL) for 30 min, mounted in McIlvaine-glycerol buffer (1:1) and stored for three days. Two probes were used in the FISH procedures: (1) pTa71 clone, containing the repeating unit of the 18S-5.8S-26S rDNA from *Triticum aestivum* Linnaeus, 1753 (Gerlach and Bedbrook 1979), and (2) pTa794 clone, which corresponds to the unit of the 5S rDNA gene isolated from *Triticum aestivum* (Gerlach and Dyer 1980). Both probes were labeled with digoxigenin-11-dUTP (Roche) by nick translation and hybridized sequentially, according to Heslop-Harrison et al. (1992). Chromosome preparations previously used in the CMA/DAPI banding were pretreated as described by Pedrosa et al. (2001). Denaturation of chromosomes and probes, post-hybridization baths and the detection of the probes were carried out as described by Heslop-Harrison et al. (1991), except for the stringency wash, which was conducted in 0.1×SSC (15 mM NaCl and 1.5 mM Na_3_C_6_H_5_O_7_.2H_2_O) at 42 °C. The hybridization mixtures consisted of 50% formamide (v/v), 10% dextran sulfate (w/v), 2×SSC and 2-5 ng/µL of the probe. The slides were denatured for 7 min at 85 °C and hybridized for at least 18 h at 37 °C. The probes were detected with a primary antibody against digoxigenin grown in sheep (DAKO) in combination with anti-sheep secondary antibody conjugated to FITC (DAKO). Slides were mounted in 2 mg/mL DAPI in Vectashield (Vector) (1:1, v/v).

Images of the best cells were captured with a Leica DMLB epifluorescence microscope with a Leica DFC 340FX camera, using the software Leica CW4000. Images were optimized for best contrast and brightness and the photos of FISH with 5S and 45 rDNA probe were pseudocolored in red and green, respectively (to allow the superposition of images), using Adobe Photoshop CS4 (Adobe Systems Incorporated). Additionally, chromosomes of 10 cells stained with DAPI of each species were measured to obtain the chromosome sizes and the relationship between the chromosome arms according to Guerra (1986), using the software UTHSCSA ImageTool, for further elaboration of the mitotic idiogram through the software Adobe Illustrator CS4 (Adobe Systems Incorporated).

## Results and discussion

**Figure 1.**
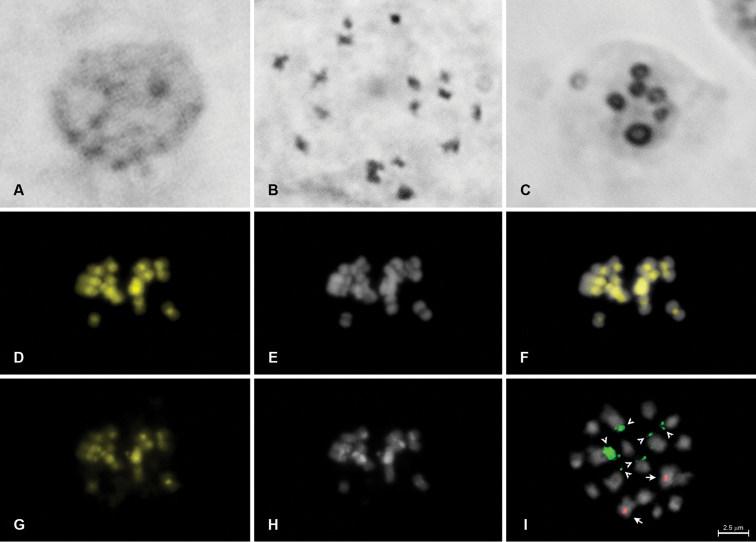
Karyotype analysis of *Euphorbia hirta* (2n = 18). Standard staining of mitotic interphase nucleus (**A**); standard staining of mitotic metaphase (**B**); silver impregnation of mitotic interphase nucleus (**C**); fluorochrome banding of metaphase chromosomes stained with CMA (**D**) and DAPI (**E**) and superposed images (**F**); C-banding of chromosomes stained with CMA/DAPI (C-CMA/DAPI; **G–H**); and metaphase chromosomes hybridized with 5S (red) and 45S (green) rDNA probes (**I**). Arrows and arrowheads indicate 5S and 45S rDNA sites, respectively.

**Figure 2.**
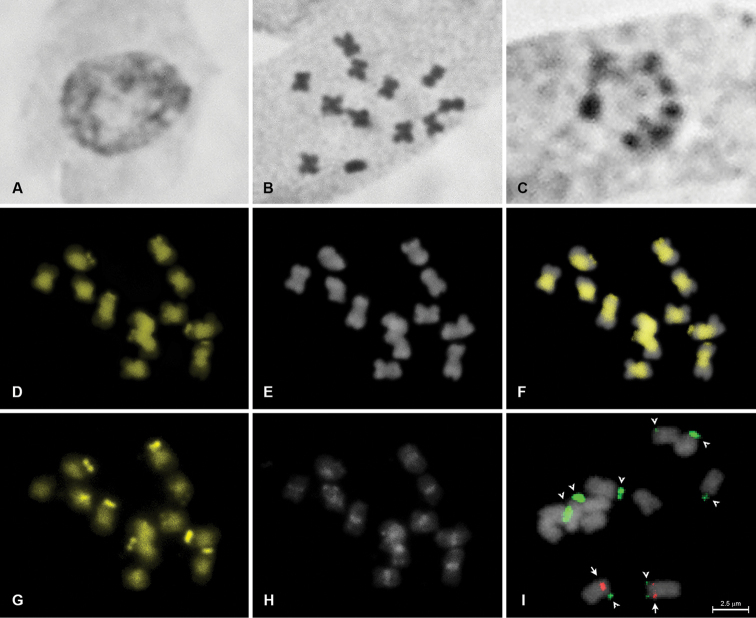
Karyotype analysis of *Euphorbia hyssopifolia* (2n = 12). Standard staining of mitotic interphase nucleus (**A**); standard staining of mitotic metaphase (**B**); silver impregnation of mitotic interphase nucleus (**C**); fluorochrome banding of metaphase chromosomes stained with CMA (**D**) and DAPI (**E**) and superposed images (**F**); C-banding of chromosomes stained with CMA/DAPI (C-CMA/DAPI; **G–H**); and metaphase chromosomes hybridized with 5S (red) and 45S (green) rDNA probes (**I**). Arrows and arrowheads indicate 5S and 45S rDNA sites, respectively.

**Figure 3.**
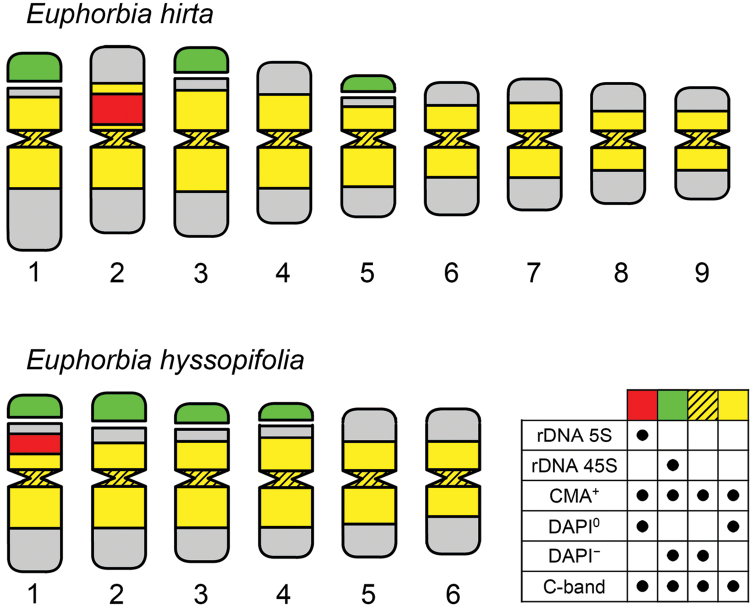
Representative idiograms of *Euphorbia hirta* and *Euphorbia hyssopifolia* chromosomes. The black dots in the chart in the inferior right corner associate the chromosome marks (rows) with their respective colors (columns) in the chromosomes.

The interphase nuclei of both species were predominantly semi-reticulated with a proximal pattern of condensation (Figures 1A, 2A). The chromosome counts showed diploid numbers of 2n = 18 for *Euphorbia hirta* and 2n = 12 for *Euphorbia hyssopifolia* (Figures 1–3), confirming previous results for both species (e.g. Wang et al. 1999, Bolaji et al. 2014). In general, the species of *Euphorbia*, in comparison with the entire family, are relatively well represented in the chromosome count lists of Euphorbiaceae members, with approximately 310 species of the genus (15.4%) with available chromosome numbers, which ranges from 2n = 12 (in *Euphorbia akenocarpa* Gussone, 1821 and several other species) to 2n = 120 (in *Euphorbia royleana* Gussone, 1821) (see Hans 1973, Rice et al. 2015). This becomes more evident when we take into account the available data for *Croton* Linnaeus, 1753 (Euphorbiaceae), with less than 3% of the species with chromosome numbers described (34 out of ca. 1,200 species; The Plant List 2013, Rice et al. 2015). In addition, the existence of several base numbers for *Euphorbia* (*x* = 6, 7, 8, 9, 10, etc.) indicates a great complexity of the processes of karyotype evolution and diversification within the group (Hans 1973, Rice et al. 2015). Therefore, one may notice the importance of the data published for *Euphorbia* species in improving the knowledge of the patterns of karyotype evolution within the family.

The karyotype presented metacentric and submetacentric chromosomes with gradual decreasing sizes, ranging in average from 1.21 µm to 2.58 µm, for *Euphorbia hirta*, and from 1.43 µm to 2.04 µm, for *Euphorbia hyssopifolia*. In general, Euphorbiaceae species exhibit small chromosomes (see Hans 1973, Vanzela et al. 1997, Carvalho and Guerra 2002), such as those found in the two species analyzed, although there is also a wide variation in chromosome sizes within the genus *Euphorbia*, which ranges from 1-15 µm (Hans 1973). Additionally, as observed for *Euphorbia hirta* and *Euphorbia hyssopifolia*, the occurrence of only metacentric and submetacentric chromosomes was already reported for other species of the family, such as *Manihot* spp. (Carvalho and Guerra 2002), *Jatropha curcas* Linnaeus, 1753 (Carvalho et al. 2008) and castor (*Ricinus communis* Linnaeus, 1753) (Vasconcelos et al. 2010). On the other hand, acrocentrics and subtelocentrics were described for other *Euphorbia* species, such as *Euphorbia characias* Linnaeus, 1753 and *Euphorbia meloformis* Aiton, 1789 (Vosa and Bassi 1991, D’Emerico et al. 2003).

*Euphorbia hirta* presented a smaller genome size (2C = 0.77 ± 0.02 pg) than *Euphorbia hyssopifolia* (2C = 1.41 ± 0.04 pg). These results fit in the known range of DNA content of species of the genus, which varies from 2C = 0.70 pg to 2C = 18.80 pg (see Bennett and Leitch 2012). According to the most comprehensive phylogenetic reconstruction based on nuclear and plastid sequences for Euphorbia subgenus Chamaesyce Gray, 1821, provided by Yang and Berry (2011), three major clades (Acuta, Hypercifolia and Peplis) were strongly supported. Thus, despite the inclusion of both *Euphorbia hirta* and *Euphorbia hyssopifolia* in the clade Hypericifolia, they were not recovered as closely related species. Therefore, a plausible explanation for the discrepancy regarding the genome size and chromosomes numbers between these two species could be their particular evolutionary histories.

However, despite accounting for more than half of the known genome sizes of members of Euphorbiaceae (19 out of 33 analyzed species), the proportion of *Euphorbia* analyzed species is considerably low, being less than 1% of the genus. Thus, the noteworthy range of variation of 41× among the species of the genus analyzed so far, between the diploid species *Euphorbia peplus* Linnaeus, 1758 (2n = 22; 2C = 0.70 pg) and *Euphorbia polygona* Haworth, 1803 (2*n* = 20; 2C = 28.70), although quite high, may be an underestimation (see Bennet and Leitch 2016). Also, while this is the first report of the genome size of *Euphorbia hyssopifolia*, Bennett et al. (1998) observed 2C = 1.30 pg for *Euphorbia hirta*, which is almost twice the value obtained here. Similarly, divergent genome sizes have been estimated for other *Euphorbia* species, such as *Euphorbia amygdaloides* L. with 2C = 5.48 pg (Vidic et al. 2009) and 2C = 7.02 pg (Temsch et al. 2010) and *Euphorbia pulcherrima* Willdenow ex Klotzsch, 1834 with 2C = 2.60 pg (Galbraith et al. 1983) and 2C = 3.30 pg (Bennet et al. 2000). In this regard, these differences in the genome size within the same species may be associated with intraspecific variation in the abundance and distribution of genomic repeat classes, such as transposable elements (Kidwell 2002, Heslop-Harrison 2012).

In the CMA/DAPI banding, the pericentromeric region of all chromosomes showed positive bands for CMA and were negative for DAPI (CMA^+^/DAPI^−^) for both species (Figures 1D–F, 2D–F), as previously observed in castor (Vasconcelos et al. 2010) and frequently reported for angiosperms with small chromosomes (Guerra 2000), such as cowpea (*Vigna unguiculata* Linnaeus, 1753) (Bortoleti et al. 2012). On the other hand, all chromosomes of both species presented neutral regions for both fluorochromes in the remaining portions of all chromosomes, except for the satellites, which were CMA^+^/DAPI^−^ (Figure 3). The Giemsa C-banding revealed the same heterochromatic regions evidenced by CMA/DAPI, which were sensibly enhanced by the CMA/DAPI/C-banding approach (Figures 1G–H, 2G–H, 3). Furthermore, after the CMA/DAPI/C-banding, DAPI bands could be observed in the pericentromeric region of all chromosomes of *Euphorbia hirta* and *Euphorbia hyssopifolia*, which may not necessarily be related to AT-rich heterochromatin, but to heterochromatin in general (Barros e Silva and Guerra 2010).

The FISH procedures revealed 45S rDNA terminal sites for both *Euphorbia hirta* (short arm of chromosome pairs 1, 3 and 5) and *Euphorbia hyssopifolia* (short arm of chromosome pairs 1, 2, 3 and 4) (Figures 1I, 2I and 3), which were always associated with CMA^+^/DAPI^−^ bands (Figure 3), such as in all previously analyzed Euphorbiaceae species (see Leitch et al. 1998, Carvalho and Guerra 2002, Vasconcelos et al. 2010). On the other hand, only one site of 5S rDNA was observed in an interstitial position of the short arm of chromosome pair 2 of *Euphorbia hirta* (Figure 1I) and pair 1 of *Euphorbia hyssopifolia* (Figure 2I), as described for all other karyotypes within the family (Leitch et al. 1998, Carvalho and Guerra 2002, Witkowska et al. 2009, Vasconcelos et al. 2010). In addition, both species presented 5S rDNA sites associated with CMA^+^ bands (Figure 3), as described for castor, in which there was also a chromosome pair bearing both 5S rDNA and 45S rDNA (Vasconcelos et al. 2010), and other few angiosperms (e.g. Cabral et al. 2006, Bernardes et al. 2013). As discussed by Roa and Guerra (2015), the occurrence of 5S and 45S rDNA sites in the same chromosome (as observed for *Euphorbia hyssopifolia*) has been reported in several angiosperm species, possibly as a consequence of random transpositions of both sequences. Additionally, the authors observed that this association is more likely to be observed when there are multiple rDNA sites, as found in the present work.

The maximum number of nucleoli per interphase nuclei visualized through impregnation with silver nitrate in *Euphorbia hirta* and *Euphorbia hyssopifolia* were six and eight, respectively (Figure 1C, 2C). However, the vast majority of cells of *Euphorbia hirta* and *Euphorbia hyssopifolia* exhibited, respectively, one (54.04%) and two (40.19%) nucleoli, with only 0.24% and 0.12% showing the maximum number of active NORs (Table 1). The low frequency of cells displaying the maximum number of evident nucleoli in both species is quite common for species with more than one NOR (see Vasconcelos et al. 2010 and references within), which probably occurred due to fusion of nucleoli and/or absence of activation of certain NORs in the previous interphase (Pikaard 2000, Preuss and Pikaard 2007).

**Table 1.** Nucleolar frequency by interphase nucleus in mitotic cells of *Euphorbia hirta* and *Euphorbia hyssopifolia*.

Species	Nucleoli per cell						Number of cells
	1 (%)	2 (%)	3 (%)	4 (%)	5–6 (%)	7–8 (%)	
*Euphorbia hirta*	54.04	34.25	9.89	1.31	0.52	-	5054
*Euphorbia hyssopifolia*	34.72	40.19	19.35	4.91	0.72	0.12	4280

It is interesting to note that despite similarities in the morphology, habit and occurrence of both here studied species in the sampled area, no evidence of hybridization was detected during field work. This was confirmed by a recent report using phytochemical profiling and ISSR (Inter-Simple Sequence Repeat) markers, positioning both taxa in distinct branches (Santana et al. 2015). Both species are sometimes confounded by the local herbal sellers, being sold under identical common names (e.g. *erva-de-santa-luzia*; Santana et al. 2015). Thus, the here observed cytogenetic features reassure their position as completely distinct taxonomic entities.

## Conclusions

The present analysis characterized the chromosomes of two *Euphorbia* species, being a pioneer in the application of the FISH methodology with members of the subfamily Euphorbioideae. The physical mapping of repetitive DNA played a complementary role between the different methodologies employed, generating markers that showed a relatively high conservation of the distribution pattern of heterochromatin between *Euphorbia hirta* and *Euphorbia hyssopifolia*. These findings indicated the high potential of the employed approaches in describing chromosome markers that may be very helpful differentiate species and understand karyotype evolution within such a diverse genus.

## References

[B1] Alisi CS Abanobi SE (2012) Antimicrobial properties of *Euphorbia hyssopifolia* and *Euphorbia hirta* against pathogens complicit in wound, typhoid and urinary tract infections. International Journal of Tropical Disease and Health 2: 72–86. doi: 10.9734/IJTDH/2012/1050

[B2] Amorozo MCM (2002) Uso e diversidade de plantas medicinais em Santo Antonio do Leverger, MT, Brasil. Acta Botanica Brasilica 16: 189–203. doi: 10.1590/S0102-33062002000200006

[B3] Araújo SS Almeida PM Randau KP Fernandes TCC Marin-Morales MA Benko-Iseppon AM Cardona YT Santos AV Brasileiro-Vidal AC (2015) Cytotoxic and genotoxic effects of ethanolic extract of *Euphorbia hyssopifolia* L. on HepG2 cells. Journal of Ethnopharmacology 170: 16–19. doi: 10.1016/j.jep.2015.04.044 25937254 10.1016/j.jep.2015.04.044

[B4] Ayyanar M Ignacimuthu S (2009) Herbal medicines for wound healing among tribal people in Southern India: ethnobotanical and scientific evidences. International Journal of Applied Research in Natural Products 2: 29–42.

[B5] Barros e Silva AE Guerra M (2010) The meaning of DAPI bands observed after C-banding and FISH procedures. Biotechnic and Histochemistry 85: 115–125. doi: 10.3109/10520290903149596 19657781 10.1080/10520290903149596

[B6] Benko-Iseppon AM Morawetz W (2000) Viburnales: cytological features and a new circumscription. Taxon 49: 5–16. doi: 10.2307/1223927

[B7] Bennett MD Leitch IJ Hanson L (1998) DNA amounts in two samples of angiosperm weeds. Annals of Botany 82: 121–134. doi: 10.1006/anbo.1998.0785

[B8] Bennet MD Bhandol P Leitch IJ (2000) Nuclear DNA amounts in Angiosperms and their modern uses – 807 new estimates. Annals of Botany 86: 859–909. doi: 10.1006/anbo.2000.1253

[B9] Bennett MD Leitch IJ (2016) Plant DNA C-values database (Release 6.0, Dec 2012). http://data.kew.org/cvalues/ [accessed 10 April 2016]

[B10] Bernardes ECS Benko-Iseppon AM Vasconcelos S Carvalho R Brasileiro-Vidal AC (2013) Intra- and interspecific chromosome polymorphisms in cultivated *Cichorium* L. species (Asteraceae). Genetics and Molecular Biology 36: 357–364. doi: 10.1590/S1415-47572013005000025 24130443 10.1590/S1415-47572013005000025 PMC3795171

[B11] Bolaji AO Olojede CB Famurewa AA Faluyi JO (2015) Morphological and cytological studies of *Euphorbia hyssopifolia* L. and *Euphorbia heterophylla* L. from Ile-Ife, Nigeria. Nigerian Journal of Genetics 28: 15–18. doi: 10.1016/j.nigjg.2015.06.003

[B12] Bortoleti KCA Benko-Iseppon AM Melo NF Brasileiro-Vidal AC (2012) Chromatin differentiation between *Vigna radiata* (L.) R. Wilczek and *V. unguiculata* (L.) Walp. (Fabaceae). Plant Systematics and Evolution 298: 689–693. doi: 10.1007/s00606-011-0551-y

[B13] Bruyns PV Mapaya RJ Hedderson T (2006) A new subgeneric classification for *Euphorbia* (Euphorbiaceae) in southern Africa based on ITS and *psb*A-*trn*H sequence data. Taxon 55: 397–420. doi: 10.2307/25065587

[B14] Cabral JS Felix LP Guerra M (2006) Heterochromatin diversity and its co-localization with 5S and 45S rDNA sites in chromosomes of four *Maxillaria* species (Orchidaceae). Genetics and Molecular Biology 29: 659–664. doi: 10.1590/S1415-47572006000400015

[B15] Carvalho CR Clarindo WR Praça MM Araújo FS Carels N (2008) Genome size, base composition and karyotype of *Jatropha curcas* L. an important biofuel plant. Plant Science 174: 613–617. doi: 10.1016/j.plantsci.2008.03.010

[B16] Carvalho R Guerra M (2002) Cytogenetics of *Manihot esculenta* Crantz (cassava) and eight related species. Hereditas 136: 159–168. doi: 10.1034/j.1601-5223.2002.1360212.x 12369103 10.1034/j.1601-5223.2002.1360212.x

[B17] D’Emerico S Pignone D Vita F Scrugli A (2003) Karyomorphological analyses and chromatin characterization by banding techniques in *Euphorbia characias* L. and *E. wulfenii* Hoppe (= *E. veneta* Willd.) (Euphorbiaceae). Caryologia 56: 501–508. doi: 10.1080/00087114.2003.10589363

[B18] Dolezel J Binarova P Lucretti S (1989) Analysis of nuclear DNA content in plant cells by flow cytometry. Biologia Plantarum 31: 113–120. doi: 10.1007/BF02907241

[B19] Dorsey BL Haevermans T Aubriot X Morawetz JJ Riina R Steinmann VW Berry PE (2013) Phylogenetics, morphological evolution, and classification of Euphorbia subgenus Euphorbia. Taxon 62: 291–315. doi: 10.12705/622.1

[B20] Gerlach WL Bedbrook JR (1979) Cloning and characterization of ribosomal RNA genes from wheat and barley. Nucleic Acids Research 7: 1869–1885. doi: 10.1093/nar/7.7.1869 537913 10.1093/nar/7.7.1869 PMC342353

[B21] Galbraith DW Harkins KR Maddox JM Ayres NM Sharma DP Firoozabady E (1983) Rapid flow cytometric analysis of the cell cycle in intact plant tissues. Science 220: 1049–1051. doi: 10.1126/science.220.4601.1049 17754551 10.1126/science.220.4601.1049

[B22] Gerlach WL Dyer TA (1980) Sequence organization of the repeated units in the nucleus of wheat, which contains 5S rDNA genes. Nucleic Acids Research 8: 4851–4865. doi: 10.1093/nar/8.21.4851 7443527 10.1093/nar/8.21.4851 PMC324264

[B23] Guerra M (1986) Reviewing the chromosome nomenclature of Levan et al. Brazilian Journal of Genetics 9: 741–743.

[B24] Guerra M (2000) Patterns of heterochromatin distribution in plant chromosomes. Genetics and Molecular Biology 23: 1029–1041. doi: 10.1590/S1415-47572000000400049

[B25] Guerra M (2012) Cytotaxonomy: the end of childhood. Plant Biosystems 146: 703–710.

[B26] Guerra M Souza MJ (2002) Como observar cromossomos: um guia de práticas em citogenética vegetal, animal e humana. Editora Funpec, Ribeirão Preto, 131 pp.

[B27] Hans AS (1973) Chromosomal conspectus of the Euphorbiaceae. Taxon 22: 591–636. doi: 10.2307/1218637

[B28] Heslop-Harrison JS (2012) Genome evolution: extinction, continuation or explosion? Current Opinion in Plant Biology 15: 115–121. doi: 10.1016/j.pbi.2012.03.006 22465161 10.1016/j.pbi.2012.03.006

[B29] Heslop-Harrison JS Schwarzacher T Anamthawat-Jonsson K Leitch AR Shi M Leitch IJ (1991) In situ hybrid with automated chromosome denaturation. Technique 3: 109–115.

[B30] Heslop-Harrison JS Harrison GE Leitch IJ (1992) Reprobing of DNA: DNA in situ hybridization preparations. Trends in Genetics 8: 372–373. doi: 10.1016/0168-9525(92)90287-E 1440872 10.1016/0168-9525(92)90287-e

[B31] Horn JW van Ee BW Morawetz JJ Riina R Steinmann VW Berry PE Wurdack KJ (2012) Phylogenetics and the evolution of major structural characters in the giant genus *Euphorbia* L. (Euphorbiaceae). Molecular Phylogenetics and Evolution 63: 305–326. doi: 10.1016/j.ympev.2011.12.022 22273597 10.1016/j.ympev.2011.12.022

[B32] Huang L Chen S Yang M (2012) *Euphorbia hirta* (Feiyangcao): a review on its ethnopharmacology, phytochemistry and pharmacology. Journal of Medicinal Plants Research 6: 5176–5185.

[B33] Kidwell MG (2002) Transposable elements and the evolution of genome size in eukaryotes. Genetica 115: 49–63. doi: 10.1023/A:1016072014259 12188048 10.1023/a:1016072014259

[B34] Kuta FA Damisa D Adamu A Nwoha E Bello IM (2014) Antibacterial activity of *Euphorbia hirta* against *Streptococcus pneumoniae*, *Klebsiella pneumoniae* and *Proteus vulgaris*. Bayero Journal of Pure and Applied Sciences 6: 65–68. doi: 10.4314/bajopas.v6i2.14

[B35] Leitch AR Lim KY Leitch IJ O’Neill M Cheye M Low F (1998) Molecular cytogenetic studies in rubber, *Hevea brasiliensis* Muell. Arg. (Euphorbiaceae). Genome 41: 464–467. doi: 10.1139/g98-012

[B36] Loureiro J Rodriguez E Dolezel J Santos C (2007) Two new nuclear isolation buffers for plant DNA flow cytometry: a test with 37 species. Annals of Botany 100: 875–888. doi: 10.1093/aob/mcm152 17684025 10.1093/aob/mcm152 PMC2749623

[B37] Mwine TJ Van Damme P (2011) Why do Euphorbiaceae tick as medicinal plants? – a review of Euphorbiaceae family and its medicinal features. Journal of Medicinal Plants Research 5: 652–662.

[B38] Pedrosa A Jantsch MF Moscone EA Ambros PF Schweizer D (2001) Characterisation of pericentromeric and sticky intercalary heterochromatin in *Ornithogalum longibracteatum* (Hyacinthaceae). Chromosoma 110: 203–213. doi: 10.1007/s004120000125 11513295 10.1007/s004120000125

[B39] Pikaard CS (2000) The epigenetics of nucleolar dominance. Trends in Genetics 16: 495–500. doi: 10.1016/S0168-9525(00)02113-2 11074291 10.1016/s0168-9525(00)02113-2

[B40] Preuss S Pikaard CS (2007) rRNA gene silencing and nucleolar dominance: insights into a chromosome-scale epigenetic on/off switch. Biochimica et Biophysica Acta 1769: 383–392. doi: 10.1016/j.bbaexp.2007.02.005 17439825 10.1016/j.bbaexp.2007.02.005 PMC2000449

[B41] Rice A Glick L Abadi S Einhorn M Kopelman NM Salman-Minkov A Mayzel J Chay O Mayrose I (2015) The chromosome counts database (CCDB) – a community resource of plant chromosome numbers. New Phytologist 206: 19–26. doi: 10.1111/nph.13191 25423910 10.1111/nph.13191

[B42] Roa F Guerra M (2015) Non-random distribution of 5S rDNA sites and its association with 45S rDNA in plant chromosomes. Cytogenetic and Genome Research 146: 243–249. doi: 10.1159/000440930 26489031 10.1159/000440930

[B43] Santana KCB Pinangé DSB Randau KP Alves MVS Benko-Iseppon AM (2015) The medicinal species *Euphorbia hirta* and *E. hyssopifolia* (Euphorbiaceae): uses, phytochemical data and genetic diversity. In: Duarte MCT Rai M (Eds) Therapeutic medicinal plants: from lab to the market. Boca Raton, 154–173. doi: 10.1201/b19773-9

[B44] Schneider AA (2007) A flora naturalizada no Estado do Rio Grande do Sul, Brasil: herbáceas subespontâneas. Biociências 15: 257–268.

[B45] Schweizer D Ambros PF (1994) Chromosome banding. In: Gosden JR (Ed.) Methods in molecular biology, 29 Humana Press, Totowa, 97–112. 10.1385/0-89603-289-2:97 7518289

[B46] Shi QW Su XH Kiyota H (2008) Chemical and pharmacological research of the plants in genus *Euphorbia*. Chemical Reviews 108: 4295–4327. doi: 10.1021/cr078350s 18817355 10.1021/cr078350s

[B47] Steinmann VW Porter JM (2002) Phylogenetic relationships in Euphorbieae (Euphorbiaceae) based on ITS and *ndh*F sequence data. Annals of Missouri Botanical Gardens 89: 453–490. doi: 10.2307/3298591

[B48] Temsch EM Temsch W Ehrendorfer-Schratt L Greilhuber J (2010) Heavy metal pollution, selection and genome size: the species of the Zervaj study revisited with flow cytometry. Journal of Botany 2010: 1–11. doi: 10.1155/2010/596542

[B49] The Plant List (2013) Version 1.1 http://www.theplantlist.org [accessed 10 September 2015]

[B50] Vanzela ALL Ruas PM (1997) Karyotype studies of some species of *Dalechampia* Plum. (Euphorbiaceae). Botanical Journal of the Linnean Society 125: 25–33. doi: 10.1111/j.1095-8339.1997.tb02244.x

[B51] Vasconcelos S Souza AA Gusmão CLS Milani M Benko-Iseppon AM Brasileiro-Vidal AC (2010) Heterochromatin and rDNA 5S and 45S sites as reliable cytogenetic markers for castor bean (*Ricinus communis*, Euphorbiaceae). Micron 41: 746–753. doi: 10.1016/j.micron.2010.06.002 20615717 10.1016/j.micron.2010.06.002

[B52] Vidic T Greilhuber J Vilhar B Dermastia M (2009) Selective significance of genome size in a plant community with heavy metal pollution. Ecological Applications 19: 1515–1521. doi: 10.1890/08-1798.1 19769099 10.1890/08-1798.1

[B53] Vieira R Queiroz A Morais L Barao A Mello-Sampayo T Viegas W (1990) Genetic control of 1R nucleolus organizer region expression in the presence of wheat genomes. Genome 33: 713–718. doi: 10.1139/g90-107

[B54] Vosa CG Bassi P (1991) Chromosome studies in the Southern African flora. The basic karyotype of eight species of succulent *Euphorbia* L. Caryologia 44: 27–33. doi: 10.1080/00087114.1991.10797016

[B55] Wang Y Ma J Liu Q (1999) Karyotypes of eight species of *Euphorbia* L. (Euphorbiaceae) from China. Acta Phytotaxonomica Sinica 37: 394–402.

[B56] Webster GL (1994) Classification of the Euphorbiaceae. Annals of the Missouri Botanical Garden 81: 3–32. doi: 10.2307/2399908

[B57] Webster GL (2014) Euphorbiaceae. In: Kubitzki K (Ed.) The families and genera of vascular plants. Volume 11. Malpighiales. Heidelberg, New York, Dordrecht, London, 51–216. doi: 10.1007/978-3-642-39417-1_10

[B58] Witkowska M Ohmido N Cartagena J Shibagaki N Kajiyama S Fukui K (2009) Physical mapping of ribosomal DNA genes on *Jatropha curcas* chromosomes by multicolor FISH. Cytologia 74: 133–139. doi: 10.1508/cytologia.74.133

[B59] Yang Y Berry PE (2011) Phylogenetics of the *Chamaesyce* clade (*Euphorbia*, Euphorbiaceae: reticulate evolution and long-distance dispersal in a proeminent C4 lineage. American Journal of Botany 98: 1486–1503. doi: 10.3732/ajb.1000496 21875975 10.3732/ajb.1000496

